# Systematic review of the application of the Kidney Failure Risk Equation and Oxford classification in estimating prognosis in IgA Nephropathy

**DOI:** 10.1186/s13643-024-02739-2

**Published:** 2025-01-16

**Authors:** M. P. Toal, R. Fergie, M. P. Quinn, C. J. Hill, C. O’Neill, A. P. Maxwell

**Affiliations:** 1 Centre for Public Health, Queen’s University of Belfast, Belfast, BT12 6AB Northern Ireland; 2 Regional Nephrology and Transplant Unit, Belfast City Hospital, Lisburn Road, BT9 7BA Belfast, Northern Ireland

## Abstract

**Background:**

IgA nephropathy (IgAN) is the most common primary glomerulonephritis in the world and is an important cause of chronic kidney disease (CKD) and kidney failure. Outcomes are heterogeneous, and accurate risk stratification is important to identify the highest risk individuals for treatment and to help prevent disease progression. The Oxford classification (OC) is an internationally adopted standard for renal biopsy reporting in IgAN, which measures the degree of histological abnormalities and predicts prognosis. The kidney failure risk equation (KFRE) was developed to predict kidney failure in all causes of CKD and has been shown to be highly accurate across diverse etiologies. This review aimed to compare the KFRE with formulae incorporating the OC in accurately determining the risk of kidney failure in IgAN.

**Methods:**

A systematic review was conducted in accordance with the Cochrane library guidelines and PRISMA statement for reporting of systematic reviews. Studies comparing the accuracy of the KFRE with the OC in predicting disease progression and kidney failure in IgAN were evaluated. The search strategy and analysis were performed independently by two reviewers. Studies that were eligible for inclusion compared the KFRE with any tool incorporating the OC in a cohort of individuals with IgAN. Eligible outcomes were reduction of estimated glomerular filtration rate (eGFR) or end-stage renal disease (ESRD), and prognostic tools were required to assess the accuracy of these formulae by discrimination and/or calibration.

**Results:**

After searching several databases, only one study was eligible for inclusion in the review. This study of 2300 Chinese individuals with IgAN had a median follow-up of 2.5 years. Two-hundred eighty-eight individuals had a composite outcome of 50% decline in eGFR or ESRD, and 214 individuals developed ESRD. Both the KFRE and the IgAN Risk Prediction (IRP) tool (incorporating the OC) were highly accurate at predicting ESRD with a C-statistic of 0.90 and 0.91, respectively. Subgroup analysis demonstrated improved performance of IRP over KFRE in discrimination for individuals with preserved eGFR (> 60 ml/min/1.73 m^2^) at baseline. The risk of bias was high due to insufficient follow-up and handling of missing data, so overall confidence in findings is very low.

**Conclusion:**

There is currently insufficient evidence to compare the accuracy of the KFRE and OC in determining outcomes in IgAN. Further research is required in this field.

**Systematic review registration:**

PROSPERO CRD42022364569.

## Introduction

### Rationale

IgA nephropathy (IgAN) is the most common form of primary glomerulonephritis worldwide [1]. The course of IgAN is heterogeneous with up to 30% of affected individuals progressing to end-stage renal disease (ESRD) within 10 years, while others have stable disease without significant progression over decades [2, 3]. Identifying individuals at greatest risk for progression to ESRD is challenging, and the 2021 Kidney Disease: Improving Global Outcomes (KDIGO) guidelines highlighted risk stratification as a high priority for research in IgAN [4]. The understanding of IgAN pathophysiology is evolving, and multiple therapeutic trials are ongoing to find novel agents to stabilize patients at the highest risk of progression to ESRD [5]. Previous therapeutic trials in IgAN are arguably of limited utility because they recruited many individuals at lower risk for progression to ESRD [6]. Earlier risk stratification to identify persons most likely to progress to ESRD would enable more targeted clinical trials in the future [6, 7].

The kidney failure risk equation (KFRE) (Table 1) was developed from a Canadian cohort and validated in a multinational cohort of over 700,000 patients [8]. This equation, using either four or eight variables, has demonstrated excellent discrimination and can accurately predict the risk of kidney failure at a 2- or 5-year horizon for all forms of chronic kidney disease (CKD). The IgAN risk prediction (IRP) tool was developed specifically for IgAN and incorporates the Oxford classification (OC) into the risk evaluation (Table 1) [9]. OC is a histopathological scoring system developed to aid prognostic estimations by classifying the degree of pathological abnormalities demonstrated on renal biopsy examination, specifically for IgAN [10]. A renal biopsy is a crucial investigation in nephrology which facilitates the diagnosis of kidney diseases and estimation of prognosis and guides treatment [11]. It is however an invasive procedure, with a significant risk of bleeding, requiring intervention in around 1 in 200 cases [12, 13]. While both the KFRE and IRP/OC have been externally validated, there is limited evidence to determine which method provides optimal prognostic assessment and if undergoing a renal biopsy improves the accuracy of this estimation. Affected individuals will often have multiple blood and urine tests over a period of follow-up; however, an invasive renal biopsy is seldom repeated. The OC will therefore provide a snapshot of disease activity, but does not capture the nuances and dynamic changes in disease trajectory, which can be routinely tracked with blood and urine tests. If the OC did not provide superior predictive accuracy compared to clinical features alone, the international community may reconsider the utility of the OC in renal biopsy reports of IgAN [14].

**Table 1:** Parameters required for each prognostic algorithm

**Variable**	**KFRE**	**OC**	**IRP**
Sex	✔		
Age	✔		✔
Race			✔
Location	✔		
eGFR	✔		✔
Proteinuria^a^	✔		✔
MAP			✔
M^b^		✔	✔
E^b^		✔	✔
S^b^		✔	✔
T^b^		✔	✔
C^b^		✔	
RASB use at time of biopsy			✔
Immunosuppression use at/prior to biopsy			✔
**Outcome**	Risk of kidney failure at 2 and 5 years		Risk of composite outcome of 50% decline in eGFR and kidney failure at 5 years

*KFRE* kidney failure risk equation, *OC* Oxford classification, *IRP* IgAN Risk Prediction tool, *eGFR* estimated glomerular filtration rate (ml/min/1.73 m^2^), *MAP* mean arterial pressure (mmHg), *M* mesangial hypercellularity score, *E* endocapillary hypercellularity score, *S* segmental sclerosis score, *T* tubular atrophy/interstitial fibrosis score, *C* crescents score, *RASB* renin-angiotensin system blockers

^a^Proteinuria expressed as urinary albumin-creatinine ratio in KFRE and g/day in IRP

^b^Obtained by renal biopsy

#### Objective

Review question: Does the kidney failure risk equation provide more accurate prediction of disease progression and kidney failure than the Oxford classification (MEST-C score) in patients with IgA nephropathy?

## Methods

This study was conducted in accordance with the Cochrane library guidelines and is described in line with the PRISMA statement for reporting of systematic reviews [14, 15].

### Eligibility criteria

Studies that were considered for inclusion in the review were original cohort studies which investigated a population of patients diagnosed with IgAN on native renal biopsy and compared the prognostic accuracy of the KFRE with an alternative prediction tool which incorporates the OC of histopathological assessment. The inclusion criteria for study outcomes were progression of disease as defined by a reduction in estimated glomerular filtration rate (eGFR), rise in serum creatinine or kidney failure events as defined by an eGFR below 15 ml/min/1.73 m^2^, or use of renal replacement therapies (kidney transplantation or dialysis). Studies that included measurements of discrimination and calibration such as the area under the curve (AUC) of the receiver-operating curve or Harrell’s C-statistic were included. Studies that compared predicted outcomes with observed outcomes were also eligible for inclusion. Excluded studies were those that did not report either of the aforementioned outcomes and were limited to other parameters such as relative risk and hazard ratios without a direct measure of classification. Follow-up time of the cohort equal to or beyond 2 years from renal biopsy was considered appropriate for inclusion in the analysis.

The KFRE was first published in 2011; therefore, studies from 2011 onwards were considered for inclusion. The research subjects were humans only. Eligibility criteria were not restricted to the English language, and translation services were available if required. All funding sources were collected for included studies.

### Information sources

The following databases were searched: MEDLINE, Embase, Scopus, PubMed, Web of Science, and Google Scholar.

### Search strategy

The full search strategy applied to each database is detailed in Table 2.

**Table 2:** Search strategy applied to each database

**MEDLINE**	
Keywords, phrases, and all subheadings	1. “IgA Nephropathy”
	2. “Immunoglobulin A Nephropathy”
	3. “Glomerulonephritis, IGA”
	4. “Berger$”
	5. 1 OR 2 OR 3 OR 4
	6. “Kidney Failure Risk Equation”
	7. “KFRE”
	8. 6 OR 7
	9. “Oxford”
	10. “MEST”
	11. “MEST-C”
	12. 9 OR 10 OR 11
	13. “Disease prog$
	14. “Prognos$”
	15. “Risk$”
	16. 13 OR 14 OR 15
	17. 5 AND 8 AND 12 AND 16
**Embase**	
Keywords, phrases, and all subheadings	1. “IgA Nephropathy”
	2. “Immunoglobulin A Nephropathy”
	3. “Glomerulonephritis, IGA”
	4. “Berger$”
	5. 1 OR 2 OR 3 OR 4
	6. “Kidney Failure Risk Equation”
	7. “KFRE”
	8. 6 OR 7
	9. “Oxford”
	10. “MEST”
	11. “MEST-C”
	12. 9 OR 10 OR 11
	13. “Disease prog$
	14. “Prognos$”
	15. “Risk$”
	16. 13 OR 14 OR 15
	17. 5 AND 8 AND 12 AND 16
**Scopus**	
Article title, abstract, keywords	“IgA Nephropathy” OR “Immunoglobulin A Nephropathy” OR “Glomerulonephritis, IGA” OR “Berger$”
AND	
Article title, abstract, keywords	“Kidney Failure Risk Equation” OR “KFRE”
AND	
Article title, abstract, keywords	“Oxford” OR “MEST” OR “MEST-C”
AND	
Article title, abstract, keywords	“Disease prog$” OR “Prognos$” OR “Risk$”
**PubMed**	
All fields	“IgA Nephropathy” OR “Immunoglobulin A Nephropathy” OR “Glomerulonephritis, IGA” OR “Berger$”
AND	
All fields	“Kidney Failure Risk Equation” OR “KFRE”
AND	
All fields	“Oxford” OR “MEST” OR “MEST-C”
AND	
All fields	“Disease prog$” OR “Prognos$” OR “Risk$”
**Web of Science**	
Topic	“IgA Nephropathy” OR “Immunoglobulin A Nephropathy” OR “Glomerulonephritis, IGA” OR “Berger$”
AND	
Topic	“Kidney Failure Risk Equation” OR “KFRE”
AND	
Topic	“Oxford” OR “MEST” OR “MEST-C”
AND	
Topic	“Disease prog$” OR “Prognos$” OR “Risk$”
**Google Scholar**	
Keywords and phrases	“IgA Nephropathy” AND “Kidney Failure Risk Equation” AND “Oxford”

### Selection process

Study records were imported into reference management software (Rayyan™) which removed duplicates and maintained a library of eligible studies. Screening of studies was performed independently by the corresponding author (M. T.) and a second reviewer (R. F.). A third reviewer was available to resolve conflicts (A. P. M.), but this was not required. Studies were initially screened by title, then by abstract, and then by reviewing the full-text article.

### Data collection process

Data were extracted independently using a standardized form for collating study data by a single reviewer (M. T.). Both the first and second author used this form to collect data on the population under study, the measurement tools for prognosis, and the reported outcomes in each paper. Furthermore, both reviewers independently considered the risk of bias and confidence in findings in a standardized assessment. These evaluations remained blinded until the reviewers discussed their findings to reach a consensus position. Study authors were not contacted for additional information by the reviewers.

### Risk of bias in individual studies

Risk of bias was assessed in any study meeting the inclusion criteria using *PROBAST* (*P*rediction model *R*isk *O*f *B*ias *AS*sessment *T*ool) [16]. This tool was designed specifically for studies of prognostic assessment, as many different prediction methods can be available for single disease entities. It comprises 20 signaling questions across four domains: participants, predictors, outcomes, and analysis. For each domain, the reviewer is required to determine a risk of bias and a level of concern regarding the study’s applicability to the review question. Two independent reviewers (M. T. and R. F.) used PROBAST to assess the risk of bias, with A. P. M. acting as a third reviewer.

### Effect measures

Measurements of discrimination and calibration were collected to compare the accuracy of the prognostic tools. These included the AUC of a receiver operating curve, the C-statistic generated from this, the Akaike information criterion (AIC), and net reclassification index (NRI).

### Synthesis methods

The reviewers planned to synthesize results to allow for suitable comparison in the whole study population and subgroup analysis of similar individuals matched by age, sex, and location. The reviewers planned to express serum creatinine concentrations in µmol/l. They planned to express all measures of proteinuria in equivalent values of albumin-creatinine ratios using previously published conversion factors [17]. However, in this instance, synthesis was not possible, as only one study was included; therefore, results are described narratively.

### Reporting bias assessment

Heterogeneity and reporting bias assessment were planned but not possible due to the limited number of studies meeting inclusion criteria; however, a circumspect assessment is offered within the constraints of the available evidence.

### Confidence in cumulative evidence

The certainty in the evidence presented was analyzed using the GRADE framework. This assessment analyzes the quality of five domains: risk of bias, inconsistency, imprecision, indirectness, and publication bias [18]. This system was applied to each described outcome, detailing the reviewers’ confidence in each conclusion. This assessment was conducted independently by two reviewers (M. T. & R. F.) with a third reviewer (A. P. M.) available to resolve conflicts; however, this was not required.

## Results

### Study selection

**Fig. 1:**
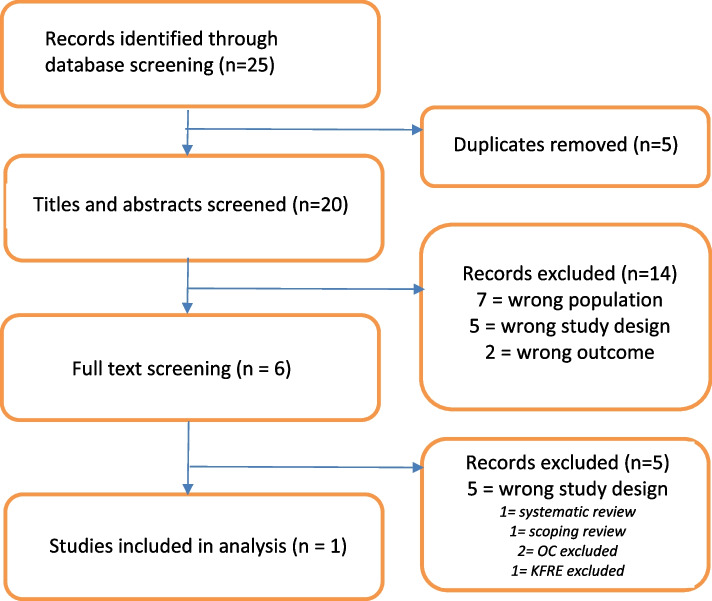
PRISMA flow chart for study selection

Twenty-five articles were identified through database searches. After duplicates were removed, 20 titles and abstracts were screened. Fourteen articles were excluded at this stage, and only 6 proceeded to full-text review. After detailed review of these articles, only one study met the pre-specified inclusion criteria (Fig. 1).

### Study characteristics

As described in the flow chart (Fig. 1), only one study met the pre-specified inclusion criteria for this review. Ouyang et al. investigated a Chinese cohort of 2300 patients with IgAN in a retrospective cohort study (Table 3) [19]. Four models of risk were evaluated: the KFRE (Tangri et al.) and the IgAN Risk Prediction tool divided into the three subtypes listed in the original derivation study (Barbour et al.): specifically the clinical model (clinical factors without OC, medication use, or race), the limited model (OC included but medication use and race excluded), and full model (OC, medication use, and race included) [8, 9]. These tools were applied to evaluate the accuracy of two renal end points: a composite outcome of 50% decline in eGFR or ESRD or ESRD alone. In this study, ESRD was defined as an *eGFR* < 15 ml/min/1.73 m^2^ or use of renal replacement therapies. A C-statistic was generated from the area under the receiver operating curve, which is a determination of how well a model discriminates between individuals who do and do not develop the outcome of interest. For this analysis, a C-statistic of 1.0 is considered a perfect model, 0.5 is equal to random chance, and greater than 0.8 is considered a good–excellent predictive model. In this instance, all models under examination demonstrated excellent discrimination at identifying those who went on to develop ESRD (Table 4) [19].

**Table 3:** Study baseline and outcome characteristics

Baseline characteristics	Ouyang et al
Country	China
No. of participants	2300
Median follow-up	2.5 years
Death (%)	10 (0.4)
Median age (IQR)	35 years (28–44)
Male (%)	1,106 (48.1%)
Creatinine at biopsy — median (IQR) µmol/l	95 (74–134)
eGFR at biopsy — median (IQR) (ml/min/1.73 m^2^)	76.9 (50.1–103.6)
MAP at biopsy — median (IQR) (mmHg)	96.3 mmHg (88.3–105.7)
Proteinuria at biopsy — median (IQR)(g/d)	1.3 (0.7–2.6)
**Primary outcome**	
50% decline in eGFR (%)	264 (11.5)
ESRD (%)	214 (9.3)
Total primary outcome (%)	288 (12.5)

*eGFR* estimated glomerular filtration rate (ml/min/1.73 m^2^), *ESRD* end-stage renal disease, *g/d* grams per day, *IQR* interquartile range, *MAP* mean arterial blood pressure (mmHg)

**Table 4:** Goodness-of-fit models for risk of ESRD at 5 years in total group and in subgroups based on baseline renal function

	*Clinical models*		*Clinical and pathology models*	
**Total** (*n* = 1764)	**Clinical model**	**CKD model**	**Limited model**	**Full model**
C-statistic	0.90 (0.86–0.93)	0.90 (0.86–0.94)	0.91 (0.88–0.94)	0.91 (0.88–0.95)
AIC	453.14	450.11	441.26	440.24
*R*^2^	0.31	0.31	0.32	0.31
**Subgroups** (C-statistic)				
eGFR < 60 (*n* = 589)	0.83 (0.78–0.88)	0.83 (0.79–0.88)	0.84 (0.80–0.89)	0.85 (0.81–0.89)
eGFR > 60 (*n* = 1175)	0.70 (0.52–0.89)	0.78 (0.62–0.95)	0.90 (0.84–0.97)	0.93 (0.87–0.99)

*ESRD* end-stage renal disease, *eGFR* estimated glomerular filtration rate (ml/min/1.73 m^2^), *AIC* Akaike information criterion. Higher values for C-statistic and *R*^2^ indicate better models. Lower values for AIC indicate better models

In subgroup analysis, there was minimal improvement with the addition of the OC for individuals with an *eGFR* < 60 ml/min/1.73 m^2^ at the time of biopsy. However, there was a considerable improvement in predictive performance for individuals with a preserved eGFR > 60 ml/min/1.73 m^2^ at the time of biopsy (Table 4). This suggests that pathological features obtained on the kidney biopsy aided risk stratification if kidney function was well preserved at the time of diagnosis; however, these features had a more limited impact on prognosis in individuals with significantly impaired kidney function.

### Risk-of-bias assessment

The risk of bias and applicability in the one included study were assessed using the PROBAST tool for prognostic modeling studies [16]. Results are summarized in Table 5. The participants who met the inclusion criteria were appropriate and consistent with other studies in the field. Therefore, the cohort under study was relevant and applicable to the review question, and both of these parameters had a low level of concern; however, the generalizability could be questioned due to the lack of ethnic diversity in this exclusively Chinese study cohort. Elements in the predictors and outcome domains raised significant concerns: the authors stated that only 1764 of 2300 participants had comprehensive clinical, laboratory, and pathological records. However, there was no explanation of how missing data was handled; therefore, it is not clear if data was imputed, or complete case analysis was utilized.

**Table 5:** Risk-of-bias assessment using PROBAST

Participants		Predictors		Outcome		Analysis	Overall	
Risk of bias	Applicability	Risk of bias	Applicability	Risk of bias	Applicability	Risk of bias	Risk of bias	Applicability
								

Low risk/level of concern.  High risk/level of concern.  Uncertain risk/level of concern

The length of follow-up is an additional concern, as IgAN typically follows a slowly progressive course over several years. Many cohort studies report 5-, 10-, or 20-year renal survival. In this study, the median length of follow-up is 2.5 years, and no interquartile range or other measure of dispersion is offered. Furthermore, the models under analysis are validated at 5-year outcomes without any explanation of how many individuals in the dataset had sufficient follow-up to conduct this analysis.

For these reasons, the overall risk of bias was determined to be high, with a low concern regarding applicability.

### Results of individual study

The study by Ouyang et al. described the goodness of fit of various models divided by two outcomes: composite of ESRD and 50% decline in eGFR at 5 years and ESRD alone. The authors present 5-year outcome data based on 1764 patients. For the composite outcome, all the presented models performed well, and there was minimal difference observed when parameters were added.

Arguably, the more meaningful outcome is ESRD at 5 years due to the clinical consequences associated with kidney failure and renal replacement therapies (RRT). The summary of the goodness-of-fit models is presented in Table 4. For the C-statistic, all models showed excellent discrimination in the total cohort; however, when patients were differentiated by their level of renal function at the time of biopsy (eGFR), there was a notable improvement in models that contained pathological variables for “low-risk” individuals with an *eGFR* > 60 ml/min/1.73 m^2^ compared to “high-risk” individuals with an *eGFR* < 60 ml/min/1.73 m^2^ at the time of biopsy.

For high-risk patients, both the CKD model (KFRE) and the full model are incorporating the OC underpredicted risk, with a higher number of observed primary outcomes than what was predicted.

### Synthesis and reporting biases

As only one study met the inclusion criteria, it was not possible to synthesize multiple studies for comparison, nor was it possible to assess for reporting bias through heterogeneity measures. Within the constraints of the single included study, there are no definite features that would suggest findings would be significantly different in this group compared to others. Assessing reporting bias is similarly challenging; however, the findings in the paper of Ouyang et al. are not sufficiently remarkable to suggest that this alone led to the publication, while similar studies reporting different outcomes would not. Therefore, one cannot conclude that there is definitive evidence of publication bias.

### Confidence assessment

A summary of the confidence in cumulative evidence is summarized in Table 6. The risk of bias was considerable, due to several factors detailed above; therefore, this lowered confidence levels. As there was only one included study, there was no inconsistency detected; however, this is impossible to assess reliably in this setting. There were no obvious factors within the included study to suggest that findings would be inconsistent with other studies, aside from the homogeneous Chinese population, as the prevalence and prognosis of IgAN differ between ethnic groups [20]. Low confidence was ascertained in imprecision as there was a poor explanation of how much missing data was present and how this was handled in the analysis.

**Table 6:** Confidence assessment in cumulative evidence using the GRADE framework

No. of studies	Certainty assessment						Effect			Certainty	Importance
	**Study design**	**Risk of bias**	**Inconsistency**	**Indirectness**	**Imprecision**	**Other considerations**	**No. of events**	**No. of individuals**	**Rate (95% CI)**		
Composite outcome of > 50% decline in eGFR or ESRD (follow-up: median 2.5 years; assessed with the following: ml/min/1.73 m^2^)											
1	Observational studies	Very serious	Not serious	Not serious	Serious	None	288	2300		⨁◯◯◯ Very low	Important
End-stage renal disease (ESRD): < 15 ml/min/1.73 m^2^ (follow-up: median 2.5 years; assessed with the following: ml/min/1.73 m^2^)											
1	Observational studies	Very serious	Not serious	Not serious	Serious	None	214	2300		⨁◯◯◯ Very low	Critical

The rating for indirectness was moderate, as the population under study was well-matched to the research question and the defined outcomes of 50% decline in eGFR and ESRD were consistent with international standards. Publication bias was difficult to assess as there was only one study included in the review. However, the reviewers determined that confidence was very low in this domain as there were no other studies for comparison.

## Discussion

### General interpretation

The two risk prediction models under examination in this review each have strengths and limitations. The KFRE is simple and uses readily available clinical parameters to predict the risk of kidney failure. Additionally, it can be applied to different forms of kidney disease [21]. A limitation is that it does not incorporate the histological changes demonstrated on renal biopsy, which are independently associated with disease progression [22, 23]. The IRP is more complex with 11 parameters but does incorporate the OC.

The OC has been validated in several international cohorts with varying degrees of predictive performance across the domains of the MEST-C score [22–28]. Furthermore, renal biopsy practice is subject to several potential biases at an individual and national level. The indications and contraindications to renal biopsy are contentious, and there is significant variability in practice within single countries based on clinician preference as well as patient factors such as socioeconomic status [29–31].

The OC is a static measurement from the time of renal biopsy; however, the KFRE can be applied at any time point during the patient’s disease course. Serial changes in OC have been demonstrated to marginally improve the prediction of long-term outcomes, but this would not be considered standard practice due to the associated risks of bleeding [32]. The IRP has been adapted to overcome this limitation using updated clinical parameters based on updated medication use, eGFR, and proteinuria at 1- and 2-year post-renal biopsy [33]. This updated formula improved predictive performance in terms of discrimination (C-statistic: 0.82 to 0.87) and calibration (integrated calibration index: 1.54 to 0.62) and addressed the underprediction of risk demonstrated in the original model.

The five articles that underwent full-text review were excluded for the following reasons. Two studies were reviews, a systematic and scoping review respectively, and therefore were not an appropriate study design for inclusion [34, 35]. In the systematic review, out of 11 included studies, only 2 incorporated the OC into their model [23, 36]. Other studies used a wide variety of pathological scoring systems which ranged from 3 to 20 levels of severity [28, 37–39].

A further two studies examined the use of the KFRE to predict a decline in eGFR over time; however, neither incorporated the OC into the study and so were excluded [21, 40]. Zhang et al. examined the impact of histological chronicity scores based on the presence and severity of four features (glomerulosclerosis, tubular atrophy, interstitial fibrosis, and arteriosclerosis) and found that this improved discrimination when added to the KFRE (*AUC* = 0.76), compared to the KFRE alone (*AUC* = 0.68) [40]. Ali et al. demonstrated that the KFRE offered excellent discrimination across multiple CKD etiologies, including glomerulonephritis; however, there was consistent underestimation of ESRD risk at 2 and 5 years [21].

The final study excluded at full-text review did not utilize the KFRE as a comparator to the OC in prognostic assessment. Yang et al. found that individuals with the highest levels of urinary matrix metalloproteinase 7 (urMMP7) had an almost threefold increase in risk (*HR* = 2.7) of composite renal event after adjustment for clinical features and OC [31].

Due to the paucity of available evidence in this area, it is challenging to use this evidence to influence clinical practice. At the present time, a kidney biopsy remains the sole method of diagnosing IgAN; therefore, the biopsy findings and diagnosis will likely be available contemporaneously. The prognostic implications of the OC remain uncertain; therefore, clinicians should interpret these findings within the context of other clinical features. Management strategies for IgAN are rapidly evolving and are beyond the scope of this review; however, identifying individuals at the highest risk with accurate prognostic tools is essential to facilitate the institution of timely and effective treatment.

### Limitations of review process

The review is limited by the dearth of research in this area, leading to the inclusion of only one paper for analysis. The search strategy and findings were verified and replicated independently; therefore, we maintain that all reasonable steps were taken to identify relevant papers. As IgAN is a rare condition, albeit a common cause of kidney disease, research into prognostic tools may have been limited. Furthermore, until recently, there were no specific treatments licensed to treat IgAN, which may have undermined the value of an accurate prognostic assessment, due to limited treatment options. In addition, the single included paper prohibited a meta-analysis, and the potential impact of publication bias could not be assessed.

### Implications of review of policy, practice, and future research

This review has highlighted a paucity of evidence in this area. IgAN is a heterogeneous disease where some individuals experience rapid progression to ESRD, while others follow a more indolent course. As new treatments have now been licensed to treat IgAN [41, 42], future research should focus on accurately identifying individuals at the highest risk of progression to ESRD in a timely manner. This could be incorporated into several strategies utilizing clinical, serological, and pathological features at multiple time points from diagnosis, as patients often live with asymptomatic, slowly progressive disease for years to decades. Artificial intelligence (AI) tools are rapidly being integrated into healthcare algorithms, and future prognostic models will likely harness AI to assist in clinical decision-making [43, 44]. In addition, the highly specific biomarkers available for many forms of glomerulonephritis may reduce the need for kidney biopsy if similar biomarkers, specific for IgAN, become available [45]. These developments will aim to ensure that the new therapeutic options are utilized effectively for individuals most likely to benefit, without subjecting low-risk patients to unnecessary treatments. It could also allow renal replacement therapies to be instituted efficiently.

## Conclusion

One study met the criteria for inclusion in this systematic review. This paper demonstrated that both the KFRE and the OC incorporated in the IRP showed good–excellent discrimination at predicting 50% decline in eGFR or ESRD at 5 years. The IRP demonstrated a statistically significant improvement in individuals with an *eGFR* > 60 ml/min/1.73 m^2^ at biopsy compared to the KFRE. However, there was a significant risk of bias in this paper; therefore, findings should be interpreted with caution. Further research is required to investigate the best risk stratification models in IgAN.

## Data Availability

All data generated or analyzed during this study is included in this published article.

## Abbreviations

AIC: Akaike information criterion
AUC: Area under the curve
CKD: Chronic kidney disease
eGFR: Estimated glomerular filtration rate
ESRD: End-stage renal disease
GRADE: Grades of Recommendation, Assessment, Development and Evaluation Working Group
KDIGO: Kidney Disease: Improving Global Outcomes
KFRE: Kidney failure risk equation
IgAN: Immunoglobulin A nephropathy
IQR: Interquartile range
IRP: IgAN Risk Prediction tool
MAP: Mean arterial pressure
MEST-C: Mesangial hypercellularity, endocapillary hypercellularity, segmental sclerosis, tubular atrophy/interstitial fibrosis, and crescents
PROBAST: Prediction model Risk of Bias Assessment tool
OC: Oxford classification
RASB: Renin-angiotensin system blockers
RRT: Renal replacement therapies
urMMP7: Urinary metalloproteinase 7
